# Cultural and Creative Product Design and Image Recognition Based on the Convolutional Neural Network Model

**DOI:** 10.1155/2022/2586042

**Published:** 2022-07-20

**Authors:** Sangyun Han, Zhifang Shi, Yongkang Shi

**Affiliations:** Hanseo University, Department of Design Convergence, Seosan 31962, Republic of Korea

## Abstract

The development in technology has resulted in the utilization of artificial intelligence systems in various fields. In this research, we are going to study cultural and creative product design and image recognition based on a convolutional neural network (CNN) model. A convolutional neural network is referred to as a type of artificial neural network (ANN) that is used to analyze visual images. Our proposed system deploys a convolutional neural network model for image recognition in the field of cultural and creative product design. Cultural and creative products are becoming more popular these days. The cultural and creative products are referred to as innovative products or innovative new product design which makes use of the cultural symbols and other cultural factors in their design. In simple words, it is the integration of culture and creativity in a new product design. The main aspect of cultural creative products is the incorporation of cultural features into a new product, thus obtaining a creative- and culture-based product. The study results have proved that CNN has provided an accuracy of 87%.

## 1. Introduction

It is necessary to transform photographs into digital matrixes before they can be processed using specialised algorithms at the beginning of processing. The primary focus of image processing is on mathematical advancements that allow for the creation of better photographs [1]. The processing of images has lately been used in a range of fields, ranging from the biological sciences to communications and remote sensing and from artistic expression to industrial design and manufacturing. The goal of image identification and classification technologies is to detect and categorise objects in photos, which is accomplished via the use of computer vision algorithms [2]. When it comes to image classification and identification, a number of difficulties come together, such as data mining, machine/deep learning, and image classification and identification, to name a few examples. Convergent fields of study include image retrieval and processing, but there are many more disciplines that have joined forces to establish this new research area [3]. Deep learning aims to develop a neural network that mimics the human brain's neuronal connections as closely as possible. An approach to data-driven learning is known as “deep learning.” As a result, progress in neuroscience research and development is inversely related to the expansion of deep learning theory, the discovery of a hierarchical structure in the human visual system [4]. Every time a new object is discovered, the exact same technique must be performed from the beginning. In terms of the general brain's information-processing capacities, we can observe that the higher we go up the information ladder, the more abstract the features become in terms of the characteristics of the individual brain [5]. This structure also reduces the number of deep-seated network components required. As a consequence, overfitting of the model is less likely to occur in the future [6]. When it comes to CNN, the local receptive field, weight sharing, and pooling layer are all crucial elements to take into account, among other things. CNN, which acts as a multilayer perceptron, makes use of local connections and weight sharing to enhance its performance and accuracy. As a result of the reduced weight number being employed, the optimization procedure is simplified, which helps to reduce the likelihood of overfitting. The network's ability to process two-dimensional photographs directly rather than relying on standard image recognition algorithms that require feature extraction and data reconstruction, and its ability to handle two-dimensional images without the need for data reconstruction, are all a result of these advantages [7]. Given the vast number of components in neural networks, they are susceptible to overfitting and need extended training periods to become fully functional. As a consequence of the statistical learning theory, this assertion is valid (also may be considered as having a layer of hidden nodes or not known as a “shallow model”; it has considerable benefits over boosting, logistic regression, and SVM). With its many hidden layers, an artificial neural network (ANN) is a kind of neural network with the unique ability to learn new qualities [8]. An ANN is a type of neural network that can learn new attributes. It is possible that unsupervised layer-by-layer DNN training, when applied to data with significant features, would be able to overcome the constraints of DNN training by better responding to the data's most significant traits and increasing data visualisation or categorization.

By using a more complicated algorithm, data show that the overall error rate for the top five has dropped to roughly 3.5 percent. According to statistics from the same ImageNet dataset 5, deep learning beats human eyesight in terms of accuracy, with an error rate of only 5.1 percent [9]. There is still a lot we do not know about photography's “black boxes,” despite the word being used often by CNN and other news organisations. The model's efficacy defies reasonable explanation, despite the fact that it is always evolving. The invention of convolutional neural networks, which are used in image processing and recognition, has allowed technology to advance tremendously in the last few years [10]. Key convolutional neural network models are found in the ImageNet dataset, which is used widely across the globe, including in America and in other nations. Compared to other image processing methods, the CNN methodology has a huge benefit since it does not need any prior picture processing. Many of the most important advancements in picture recognition technologies in recent history have been made possible by neural networks [11]. In other words, this network-based technology reduces image processing complexity to the level of a human being since it removes the need for either manual picture preprocessing or more complicated methods like feature extraction.

CNN's random technique has been used in facial recognition, educational image processing, intelligent driving, security, text recognition, user interfaces, picture searches, and intelligent homes, to mention just a few of the various possibilities [12]. The capacity of CNN's journalism to change characteristics that are low in level and limited in scope is one of its particular strengths. Because CNN uses local correlation, it can handle a broad variety of inputs, such as audio and text. CNN may be used for a wide range of purposes, from basic jobs like word tagging to more complex ones like machine translation and chatbot creation [13]. When employing a CNN for image identification, we need to test a variety of approaches since the depth of the network is always changing, restricting the generality of the network's architecture. As a consequence, we must make the tough option of selecting an approximate generic network structure depth for real-world applications [14].

If neural networks are made broadly accessible, the process of using them to recognise photographs is likely to be time-consuming and error-prone [15]. CNNs must be trained on equal amounts of data utilising various problem sets in order for them to come up with the same answer as a human brain. Constant differences in the distributions of training and test data complicate object separation for convolutional neural networks. The standard theory of image processing, which is based on convolutional neural networks (CNNs), has not yet been proven valid and thorough [16]. Since then, several other types of identification methods have been created that use databases to determine the breadth and depth of a network in order to determine the most effective parameters and optimization procedures. Exactly how the CNN's recognition effect is influenced by a person's characteristics is unknown at this time [17]. The initial state parameters of the CNN, and the optimization method utilised, must be taken into account throughout the process of learning to categorise and classify real-world photos. In general, if the validation set is more accurate than the training set, the model is underfitted [18]. Trying to get your model to perform better than the training data is known as “overfitting.” When making decisions, it is conceivable that the network may not behave in accordance with your expectations. To get the most out of the network or to gather additional data, a variety of settings must be adjusted. Despite the fact that a digital ethnic clothing library is critical for preserving and promoting ethnic clothing's cultural importance, it is still in its infancy [19]. To increase the quantity of results, it was decided to concentrate the majority of effort on one ethnic clothing database. Digital studies into ethnic clothing component extraction and analysis are thus very valuable for study and application, as are efforts to compile a common image library for minority clothes [20].

### 1.1. Motivation of the Study

In interactive control systems, computer vision is a critical component of the deep learning technological interface in creative and cultural product creation processes. In general, the interaction of deep learning technology is also dependent on the visual analytics system's adaptability. It can be used to include noninvasive human creative art by encouraging solely human creativity. Because of the impact of the complex background, accurate validation and analysis of user and cultural product design utilising real-time impact damaging cases is thought to be difficult. Human creative labour is inherently dynamic, and dealing with the interference issues needs a flexible solution. Structural image processing techniques are being developed to address the convolutional neural network model classify transmitter challenge, which includes face recognition and person identification in convolutional neural network models in unique single scenarios. Location mobility and movement patterns are used in spatial thermal imaging to investigate human creative and cultural industrial design behaviour. Motion equations are used to classify both targets and objects.

## 2. Materials and Methods

**Figure 1:**
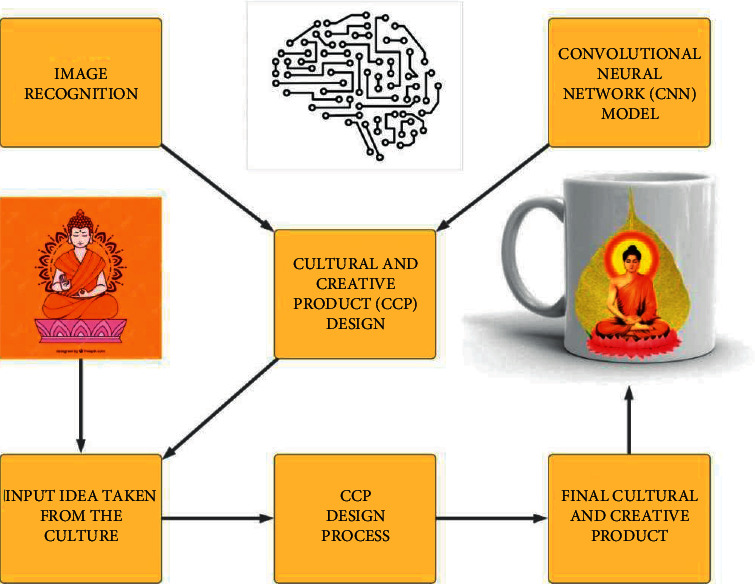
Model depicting the cultural and creative product design.

Cultural and creative products (CCPs) are becoming popular day by day. In many cultures, these types of products have a huge market especially in countries such as China and the US. These products draw their inspiration from their country's rich cultural heritage. Considering the huge market of CCP, the design of CCP is also redefined along with the growth of the technology. The cultural and creative products are referred to as innovative new product design which inspires design ideas from the culture. It can be referred as integration of culture and creativity in product design. The cultural features are incorporated into product design. The convolutional neural networks employ a mathematical operation called convolution. The neural network makes use of the convolution in place of any mathematical calculation, which is used in its layer. The convolutional layers, pooling layers, fully connected layers, hidden layers, weights, receptive fields, and visual cortex are some of the important terms used in the convolutional neural networks. In the proposed model, the convolutional neural networks (CNNs) are deployed in the image recognition process to recognise the cultural images and incorporate the idea into the new CCP design, as depicted in Figure 1. In many cases, the cultural inspirations are traditional objects such as ancient artefacts, small statues, and cultural symbols that were popular in that particular culture. The ideas were inspired by these traditional objects through the image recognition process. Image recognition is referred to as an artificial intelligence technology that identifies the different types of variables such as humans, animals, buildings, and objects in digital images. Identifying these objects is easy for human beings, whereas it is difficult for computer systems. The computer sees a digital image in the form of pixels and numeric representations. It recognizes digital images as patterns of these numerical data. The cultural and creative product (CCP) design process involves the following points: identification of a cultural model, data collection on that cultural feature, development of the design, and implementation of the design. The cultural model (traditional object) that is needed for the design process is identified at first. The data relating to that cultural model are collected, mostly by collecting the traditional objects, and in the worst scenario, the image/painting of the traditional object is used. Thus, the final cultural creative product is obtained as a finished product.

### 2.1. Convolutional Neural Network

Deep learning technology's interaction reveals a few markers of mobility or preserved disposition. The persistence of a straight line between all of these linear motions causes a proclivity for general area. The movement direction is provided by the viewpoint between the legs and the head, and the head location is known at a particular angle. *H*_1_, *H*_2_,  *H*_3_ is a human target with a running posture to the head and right slanted at an angle farther into a good direction. Its swing direction from both the head may be significant when compared to provide us with *H*_*T*_, and also the ductility as from the preliminary interactive experience, *R*_*H*_1__, *R*_*H*_1__, *R*_*H*_1__ have been magnified to focus and transcription, and also the interactive systems and the Earth's rotation between interactive systems are described as

(1) $$\begin{array}{c} H_{T_{1}}=\sum \left[R_{H_{1}},R_{H_{1}},R_{H_{1}}\right]+\sum_{n=1}^{m<p}\left[-\cos\vartheta +\sin\vartheta \right]\times \left[\sin\vartheta +\cos\vartheta \right]+\int \ln\left[n_{t}\;m_{t}\;p_{t}\right], \end{array}$$

(2) $$\begin{array}{c} H_{T_{2}}=\sum \left[R_{H_{2}},R_{H_{2}},R_{H_{2}}\right]+\sum_{n=0}^{m<p}\left[-\cos\vartheta +\sin\vartheta \right]\times \left[\sin\vartheta +\cos\vartheta \right]+\int \ln\left[n_{t}m_{t}p_{t}\right], \end{array}$$

(3) $$\begin{array}{c} H_{T_{3}}=\sum \left[R_{H_{3}},R_{3},R_{H_{3}}\right]+\sum_{n=1}^{m<p}\left[\sin\vartheta +\cos\vartheta \right]\times \left[-\cos\vartheta +\sin\vartheta \right]+\int \ln\left[n_{t}m_{t}p_{t}\right]. \end{array}$$

Equations 1–3) can be used to estimate the human physical target orientations matrix, longitudinal motion, and the translation variables of a mixing process.

Human destination needs are identified more by unit vector  *K*_*d*_, width *d*_*t*_, and height *h*_*t*_; its own ratio (*h*_*t*_1__/*d*_*t*_1__) indicates that the target *H*_1 _ is heading further towards a solitary viewpoint interactive environment represented in equation (4). The ratio (*h*_*t*_1__/*d*_*t*_1__) indicates that the target *H*_1_ is travelling towards an interaction sound system; *cs* denotes the orientation. The characters *c* and *c* − 1 represent rotation and translation in the interface design, respectively.

(4) $$\begin{array}{c} K_{d}=\sum_{y}^{c}d_{t,c}-d_{t,c-1}+\int_{t}^{c}{\left(\frac{h_{t}}{d_{t}}\right)}_{c}-\int {\left(\frac{h_{t}}{d_{t}}\right)}_{c-1}=0. \end{array}$$

These deviations have been used to denote the target *H*_1_which evolves and are therefore changed by equation for a period*t* is to determine in equation(5). The thermographic image's height *h*_*t*_ and width *d*_*t*_ are utilised to assess the technique's vector direction *K*_*d*_.

(5) $$\begin{array}{c} K_{d}=\int_{t}^{c}{\left(\frac{h_{t}}{d_{t}}\right)}_{c}-{\left(\frac{h_{t}}{d_{t}}\right)}_{c-1}\neq 0. \end{array}$$

Its alignment width is represented by *d*_*t*_, the orientation height by *h*_*t*_, and the rotation ratio by  (*h*_*t*_/*d*_*t*_). If there is no variation in sequential percentages, the direction vector *K*_*d*_ is derived from the variation in widths of a sequential objective in subsequent images.

Its overall location of extracting features for such a specific item is  *A*, and the feature extraction point for just a particular target is *x*_*f*_. The longitudinal motion feature point *n*_*f*,*c*_ is accompanied by an evaluation of its average precise location till the exact quantity of *A* is reached. The previous mean is deducted from the completed accurate location average at time *c* − 1. This variation supports the position and also the horizontally variable magnitude *b*_*t*_ from equation (6).

Condition (1) is as follows:

(6) $$\begin{array}{c} b_{t}=\underset{f⟶1}{\lim}\int_{f}^{c}\frac{\left(\sum_{f=1}^{A}n_{f,c}\right)}{A}+\sum_{t}^{c-1}d_{t,c}-d_{t,c-1}. \end{array}$$

Condition (2) is as follows:

(7) $$\begin{array}{c} b_{t}=\sum_{t}^{c}d_{t,c}-d_{t,c-1}+\underset{c-1}{\lim}\frac{\left(\sum_{f=1}^{A}n_{f,c-1}\right)}{A}. \end{array}$$

After computing,

(8) $$\begin{array}{c} b_{t}=\frac{\left(\sum_{f=1}^{A}n_{f,c-1}\right)}{A}-\underset{f⟶1}{\lim}\int_{n=1}^{A}\frac{\left(\sum_{f=1}^{A}n_{f,c}\right)}{A}, \end{array}$$

(9) $$\begin{array}{c} K_{d}=\sum \frac{\left(\sum_{f=1}^{A}n_{f,c}\right)}{A}+\sum \sqrt{K_{d}^{2}+b_{t}^{2}}\times \int_{t=1}^{c}d_{t,c}-d_{t,c-1}. \end{array}$$

As discussed in equations (7), (8), and (9),  *n*_*f*,*c*−1_ represents the feature extraction points for single target (10). The overall number of feature extraction points for a particular target is represented by  *A*. This current means is removed from the ultimate accurate location estimation at period *c* − 1. *r*. *cw* denotes the straight vector's direction and strength. *K*_*t*_ represents the final estimated route parameter.

To analyze each segment in depth, the structure is divided into *T*=(*H*_*s*_/*y*_*ver*_)(*D*_*s*_/*y*_*hor*_)  molecules. *y*_*hor*_ is the number of observations in the horizontal direction, and *y*_*ver*_ is the proportion of layers in the vertical position. The human goal is chosen for utilizing associated factors that seem to be distinct from other elements in the target frame. Equation (10) sums the value of each pixel for every cell to determine the best human target specialty for that cell.

(10) $$\begin{array}{c} {\left(nm\right)}_{u}=\sum_{n=1}^{T\in D}F_{n}+T_{n}\left(\frac{D_{b}}{y_{hor}}\right)+\int_{n=1}^{nm}f\left({\left(nm\right)}_{u}\right)\times n_{u}\left(y_{hor}\right). \end{array}$$

As noted in equation (10), *n*_*u*_ appears to be the enhanced cell scale parameter equation (11). The pixel *q* is allocated to each pixel in a cell, and the exact placements of this pixel within squares are recorded (*n*, *m*). The calculation as follows can be used to compute the final pixel directions from the underneath and left of each cell:

(11) $$\begin{array}{c} F_{t}=\sum_{n=F_{n}}^{n_{u}}\sum_{m=F_{m}}^{n_{u}}v\left(n,m\right)+\int_{n=1}^{A}\frac{\left(\sum_{f=1}^{A}n_{f,c}\right)}{A}, \end{array}$$

(12) $$\begin{array}{c} {\left(nm\right)}_{u}=\int_{n=1}^{nm}f\left({\left(nm\right)}_{u}\right)+\;m_{u}\left(y_{ver}\right)\times \sum_{m}^{\text{TH}}F_{m}+T_{m}\left(\frac{H_{s}}{y_{ver}}\right), \end{array}$$

(13) $$\begin{array}{c} n_{u}=\sum F_{m}+T_{m}\times \sum_{n=1}^{F\in T}F_{n}+T_{n}\left(\frac{D_{s}}{y_{hor}}\right)m_{u}. \end{array}$$

Equations (12) and (13) show the whole pixel coordinates of each compartment within a thermal image, where *y*_*hor*_ appears to be the quantity in the horizontal position and *y*_*ver*_ appears to be the set of nodes in the vertical orientation. *H*_*s* _ and *S*_*a*_ represent the length and size of the target frames, respectively. Its exact starting coordinates of each cell, *F*_*n*_ but also *F*_*m*_, are defined by the objective frame's current directives and also the frame shape and size are mentioned in the following equation:

(14) $$\begin{array}{c} {\left(F\right)}_{nm}=\int_{m=1}^{D\in T}\frac{D_{u}}{y_{ver}}\left(T_{m}-1\right)\times \sum_{n=1}^{m}f\left({\left(F\right)}_{nm}\right)+F_{n}\left(y_{hor}\right), \end{array}$$

(15) $$\begin{array}{c} {\left(F\right)}_{nm}=\sum_{m=1}^{H\in T}\frac{H_{u}}{y_{ver}}\left(T_{m}-1\right)\times \int_{n=1}^{m}f\left({\left(F\right)}_{nm}\right)+F_{m}\left(y_{ver}\right), \end{array}$$

(16) $$\begin{array}{c} F_{n}=\int_{n=1}^{m}f\left({\left(F\right)}_{nm}\right)+F_{n}\left(y_{hor}\right)\times \sum \frac{D_{u}}{y_{hor}}\left(T_{n}-1\right). \end{array}$$

(17) $$\begin{array}{c} F_{m}=\int_{n=1}^{m}f\left({\left(F\right)}_{nm}\right)+F_{m}\left(y_{ver}\right)\;\times \sum \frac{D_{u}}{y_{ver}}\left(T_{m}-1\right). \end{array}$$

As indicated in equations (15), (16), and (17), each mobile technology index is calculated from *T*_*m*_=[*T* − 1/*y*_*ver*_]+1  utilising the level purpose before division and also *T*_*m*_=*T* − *T*_*m*_*y*_*hor*_ After developing the translational motion for images, we can determine the connection of such biomechanics with every creative and cultural product design images of an equipment comprised. The extracorrelation result develops dynamism by integrating the human target creative and cultural product design approaches to pick the final path direction with the highest associate results.

## 3. Results and Discussion

**Figure 2:**
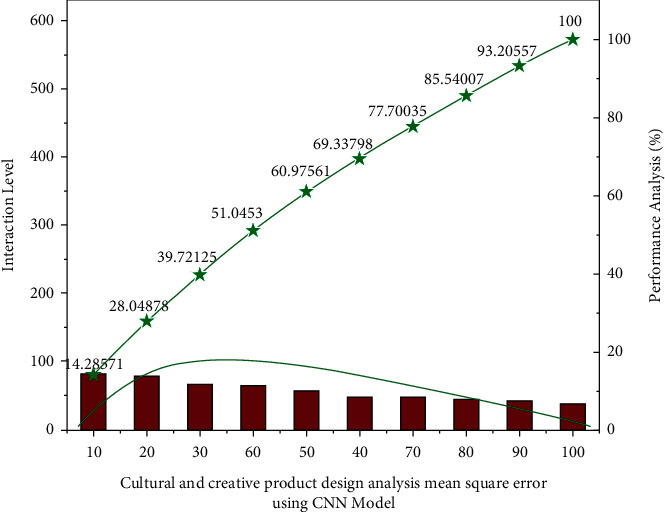
The cultural and creative product design analysis mean square error evaluated using the convolutional neural network model.

The project convolutional neural network model system's performance ratio evaluation is displayed from a certain angle. *H*_1_, *H*_2_, *H*_3_ is a human target with a running posture to the head and right slanted at an angle farther into a good direction. Its arm direction of both the head may be crucial compared to supply us with *H*_*T*_, and the ductility as from the preliminary immersive experience, *R*_*H*_1__, *R*_*H*_1__, *R*_*H*_1__ have been magnified to focus and transcribed represented in Figure 2.

To determine natural movements, the direction of a body's movement should be tracked throughout daily exercise. The use of data analysis to sufficiently demonstrate such motion aids in the identification of the activity, which also aids in the completion of this inquiry. When compared to other technologies, the effectiveness of its deep learning technology interface based on human movement detection findings of a study is extremely successful.

**Figure 3:**
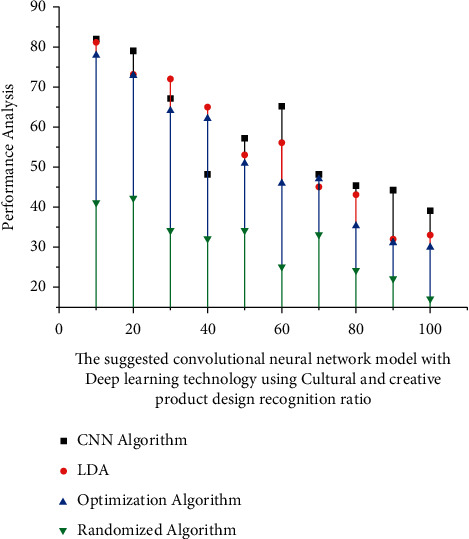
The suggested convolutional neural network model with deep learning technology using cultural and creative product design recognition ratio.

When compared to existing methodologies, the simulation results show that the proposed strategy can evaluate the human target angle with great precision. Human destination's needs are identified more by unit vector *K*_*d*_, width *d*_*t*_, and height *h*_*t*_; its own ratio (*h*_*t*_1__/*d*_*t*_1__)  indicates that the goal *H*_1 _ is moving farther towards a solitary perspective interactive environment represented in equation (2). The ratio (*h*_*t*_1__/*d*_*t*_1__)  indicates that the target *H*_1 _ is moving towards an interaction sound system; *cs* represents the orientation. In the interface design, the characters *c* and *c* − 1 denote rotation and translation, respectively, in the interface design.  Figure 3 displays the average recognition ratio attained with our suggested randomised techniques.

**Figure 4:**
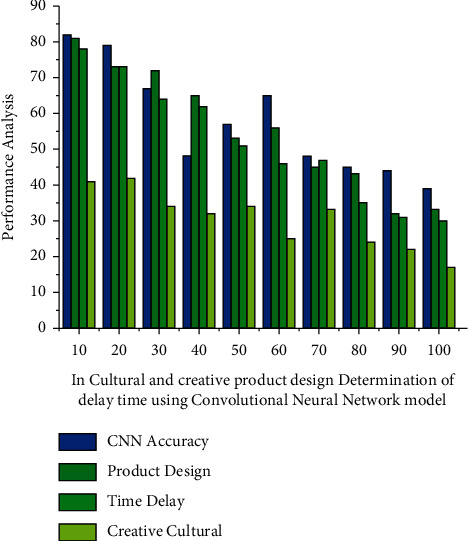
Deep learning technology in cultural and creative product design determination of delay time using the convolutional neural network model.

To analyse each segment in depth, the convolutional neural network model delay period is depicted and the structure should be divided into *T*=(*H*_*s*_/*y*_*ver*_)(*D*_*s*_/*y*_*hor*_) molecules. The number of observations in the horizontal direction is denoted by *y*_*hor*_, while the proportion of layers in the vertical direction is denoted by *y*_*ver*_. The human target is selected by using connected criteria that appear to be unique from other elements in the target frame depicted in Figure 4. The proportion of a decided design to undesirable ambient noise components demonstrates the effectiveness of a creative and cultural product design measurement. A stated design provides more information to estimate intent, improving predictive performance. However, noise from a variety of sources are available, and evaluations of the creative and cultural design processes may be polluted. An amplifier is constructed and utilised to discard or eliminate noise levels in order to maximize the transmission ratio. It was absorbed by deep learning technologies. Interaction technology is used to measure delay time in the production of creative and cultural products.

**Figure 5:**
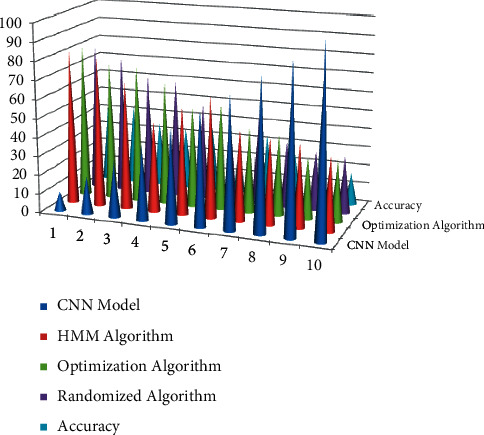
Normalised technology based on deep learning error evaluation using the convolutional neural network model in cultural and creative product design.

Potential solutions to a problem of enormous excess views were aided by design viewpoints and stereo sensing. The distance between the defined objective and the locations of an immersive experience at each degree of goal is equaled throughout a tracing cycle, depending on the operation, and is also known as the normalised error function. When compared to other established methods, the proposed strategy has a smaller computational error. The total pixel coordinates of each compartment in a thermal image, where *y* appears to be the quantity in the horizontal position and *y* is the set of nodes in the vertical position. *H*_*s*_ and *S*_*a*_ are the length and size of the target frames, respectively. The precise starting coordinates of each cell, *F*_*n*_ but also *F*_*m*_, are given by the goal frame's current commands to retrieve.  Figure 5 depicts the normalised information-processing error when the suggested convolutional neural network model technique is used.

This optimization method is far superior to the state-of-the-art linear discriminant analysis with extreme learning machine (LDA-ELM) methods, the convolutional neural network model algorithm, and the hidden Markov model with singular value decomposition (HMM-SVD) approaches. Interaction optimization, a term used to describe a technique used in deep learning, improves classification accuracy with reduced latency and noise.  Table 1 contrasts existing methods and compares our proposed algorithm using deep learning technology interaction technology in creative and cultural product design with the best outcomes offered by the randomised strategy.

In interactive control systems, computer vision is a crucial component of the deep learning technology interface in creative and cultural product development processes (shown in Table 2). The adaptability of the visual analytics system is very important in the interplay of deep learning technology. It may be utilised to foster both human creativity atomic state energy and melting of isothermal for cooling process and noninvasive art. Because of the impact of the complicated background, accurate validation and analysis of user and cultural product design utilising real-time impact damaging instances is deemed tough. Because human creative labour is essentially dynamic, a flexible solution is required to deal with interference difficulties.

## 4. Conclusion

Computer vision is an essential part of the deep learning technological interface in creative and cultural product development processes in interactive control systems. The adaptability of the visual analytics system is also critical to the interaction of deep learning technology. It can be used to encourage only human creativity while also including noninvasive art. Accurate validation and analysis of user and cultural product design using real-time impact damaging cases is considered difficult because of the impact of the complex background. A flexible solution is required to deal with interference issues because human creative labour is inherently dynamic. Face recognition and person identification in convolutional neural network models are also being addressed by structural image processing techniques, which are being developed to address the challenge of classifying transmitters. In this study, CNN technique is used to analyze the creative and cultural product design with image recognition. For future research, it is highly recommended to implement hybrid algorithm for analyzing the performance of product design using image recognition.

## Figures and Tables

**Table 1:** Interaction technology as a result of deep learning technology comparison in the design of cultural and creative products.

Number of datasets (per dataset contain 8000 data)	Convolutional neural network models (%)	LDA (%)	HMM (%)	Optimization algorithm (%)
10	87	87	79	48
20	75	79	76	43
30	69	75	65	36
40	44	68	68	35
50	53	56	52	38
60	68	53	45	27
70	46	41	43	35
80	41	47	36	28
90	48	38	30	24
100	35	35	32	18

**Table 2:** Comparison result analysis for cultural and creative product design and convolutional neural network model.

Parameter dataset	Convolutional neural network models (%)	LDA (%)	HMM (%)	Optimization algorithm (%)
Atomic state	87	87	79	48
Minimum energy	75	79	76	43
Energy	69	75	65	36
Heating to melting process	44	68	68	35
Isothermal process	53	56	52	38
Cooling process	68	53	45	27

## Data Availability

The data used to support the ﬁndings of this study are available from the corresponding author upon request.
